# Ginsenoside Rg1 Alleviates Lead-Induced Neurotoxicity Through Nrf2-Associated Modulation of Oxidative Stress and Ferroptosis

**DOI:** 10.3390/molecules31152559

**Published:** 2026-07-23

**Authors:** Yiyao Gong, Jie Zhang, Tingting Wang, Xiang Li, Hao Wang, Li Ren

**Affiliations:** 1 College of Food Science and Engineering, Jilin University, Changchun 130062, China; 2 College of Basic Medical Sciences, Jilin University, Changchun 130021, China; 3 Advanced Medical Research Institute, Shandong University, Jinan 250012, China

**Keywords:** ginsenoside Rg1, lead neurotoxicity, ferroptosis, oxidative stress

## Abstract

Chronic exposure to lead (Pb) represents a persistent environmental hazard that can impair hippocampal integrity and cognitive function, while effective protective strategies remain limited. Ginsenoside Rg1 is an important bioactive constituent derived from *Panax ginseng* and has been reported to possess antioxidant and neuroprotective activities; however, its involvement in Pb-triggered ferroptosis-associated neuronal damage remains unclear. In the present study, a male C57BL/6J mouse model of subchronic lead acetate exposure and a lead-exposed HT22 cell model were established. Behavioral and histopathological changes were assessed, followed by analysis of inflammatory responses, oxidative stress, ferroptosis-related alterations, and Nrf2-associated signaling. Rg1 improved cognitive performance and attenuated hippocampal neuronal loss, neuroinflammatory activation, oxidative injury, and ferroptosis-related alterations, including ferrous ion accumulation and mitochondrial damage. Consistently, Rg1 restored SLC7A11/xCT and GPX4 expression and enhanced Nrf2-associated antioxidant signaling, whereas the Nrf2 inhibitor ML385 weakened the Rg1-induced increases in nuclear Nrf2, NQO1, and GPX4. Collectively, these findings indicate that Rg1 alleviates lead-induced neurotoxicity and support the involvement of Nrf2 signaling in the regulation of oxidative stress and ferroptosis-related injury. These findings provide experimental evidence supporting further preclinical investigation of Rg1 and its Nrf2-associated neuroprotective mechanisms in Pb-induced neuronal injury.

## 1. Introduction

Despite decades of control measures, lead (Pb) remains a pervasive environmental toxicant because legacy contamination and ongoing industrial activities continue to sustain human exposure [1,2,3]. Its health burden is particularly evident in the nervous system, where early-life exposure can lead to long-lasting impairments in learning capacity, memory formation, and behavioral regulation [4]. In China, nationwide blood lead mapping showed that children remain vulnerable to Pb exposure, despite declining blood lead levels [5]. Even at relatively low levels, Pb exposure may affect the developing brain by entering neural tissue and disturbing neuronal homeostasis, thereby contributing to Pb-induced neurotoxicity [6].

Pb-induced neurotoxicity is closely associated with cognitive dysfunction and hippocampal injury [7,8]. Experimental studies have shown that Pb exposure impairs hippocampus-dependent learning and memory, accompanied by neuronal loss and synaptic damage in brain regions involved in cognition [7,9]. These findings provide a pathological basis for linking Pb exposure to long-term neurological impairment. Mechanistically, Pb-induced neuronal injury is partly related to oxidative stress, as excessive reactive oxygen species (ROS) can weaken endogenous antioxidant defense and disturb neuronal homeostasis [10,11,12]. Nuclear factor erythroid 2-related factor 2 (Nrf2) is a key regulator of this defensive response, and its dysfunction may increase neuronal susceptibility to toxic stress [11,12]. Recent evidence further indicates that Pb exposure can promote ferroptosis-related injury by disrupting the solute carrier family 7 member 11/glutathione peroxidase 4 (SLC7A11/GPX4) defense system, thereby connecting oxidative imbalance with regulated neuronal death [13,14]. Together, these studies suggest that effective intervention should not only reduce behavioral deficits but also preserve neuronal integrity and cellular stress resistance. More broadly, the translation of food-derived bioactive compounds into health-oriented applications has become an expanding area of food and nutrition research [15]. Experimental studies further indicate that botanical extracts and dietary bioactives can exert antioxidant, immunomodulatory, and neuroprotective effects in neuronal models [16,17].

As a representative natural product with neuroprotective potential, *Panax ginseng* has received sustained attention for its broad pharmacological activities in the nervous system [18]. Ginsenosides are the major bioactive saponins of *Panax ginseng*, with representative monomers such as Rb1, Rd, Re, Rg1, and Rg3 showing neuroprotective potential in experimental models of neural injury [19,20]. Recent evidence also indicates that ginsenosides can attenuate neuroinflammatory responses in experimental models of neurological dysfunction [21]. Among these compounds, ginsenoside Rg1 is one of the most extensively studied monomers and has attracted particular interest in neurological disease research [22,23]. Modern phytochemical and pharmacological studies have demonstrated that Rg1 exhibits prominent neuroprotective, anti-inflammatory, and antioxidant properties [24]. Rg1 has been shown to activate the Nrf2/HO-1 antioxidant axis, reduce ROS generation and neuronal apoptosis in models of cerebral ischemia–reperfusion injury [24,25]. In addition, Rg1 prevents oxidative stress-induced hippocampal neuronal apoptosis via activation of Nrf2/ARE signaling and upregulation of downstream antioxidant enzymes [24]. Although Rg1 can regulate Nrf2 signaling in several models of neural injury, its protective role under Pb exposure remains insufficiently defined. It is also unclear whether Rg1 links Nrf2-mediated antioxidant defense to ferroptosis inhibition in Pb-induced neurotoxicity. Direct pathway-specific evidence for this relationship is still lacking.

On the basis of these observations, we hypothesized that Nrf2 signaling contributes to the protective effects of ginsenoside Rg1 against lead-induced neuronal injury and ferroptosis-related damage. To address this hypothesis, we combined behavioral evaluation, hippocampal histological analysis, biochemical assays, ferroptosis-related measurements, and ML385-based pharmacological inhibition. These approaches were used to assess cognitive function, neuronal integrity, oxidative and inflammatory responses, xCT/GPX4 defense, and Nrf2 signaling under Pb exposure. This study aimed to evaluate the involvement of Nrf2 signaling in Rg1-mediated neuroprotection and to provide experimental evidence for the protective potential of Rg1 against Pb-associated neurotoxicity.

## 2. Results

### 2.1. Rg1 Ameliorated Pb-Induced Cognitive Impairment in Mice

**Figure 1:**
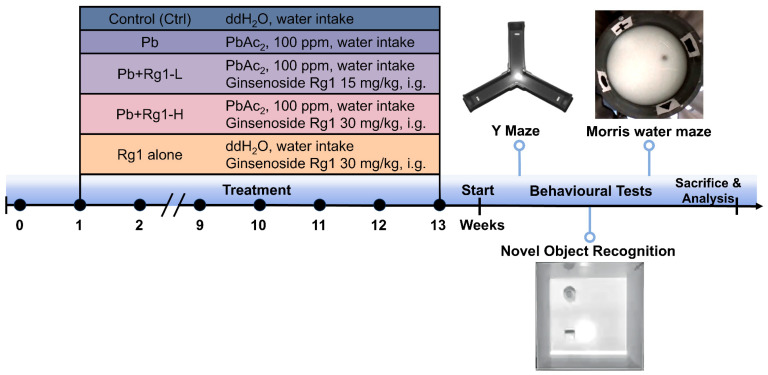
Experimental design used to assess the influence of subchronic exposure to Pb and ginsenoside Rg1 intervention on cognitive function in mice. Following a 1-week acclimation period, the mice were randomly assigned to five groups. These included the Ctrl group administered ddH_2_O; the Pb model group exposed to 100 ppm PbAc_2_ through drinking water; the Pb + Rg1-L and Pb + Rg1-H groups orally gavaged with Rg1 at 15 and 30 mg/kg, respectively; and the Rg1-alone group gavaged with 30 mg/kg Rg1. After the 12-week intervention, behavioral assessments were conducted sequentially without overlap. The Y-maze was performed first, followed by the NOR assay and the 7-day MWM procedure. Thereafter, animals were euthanized, and brain tissues were harvested for further biochemical analyses and histopathological evaluation.

**Figure 2:**
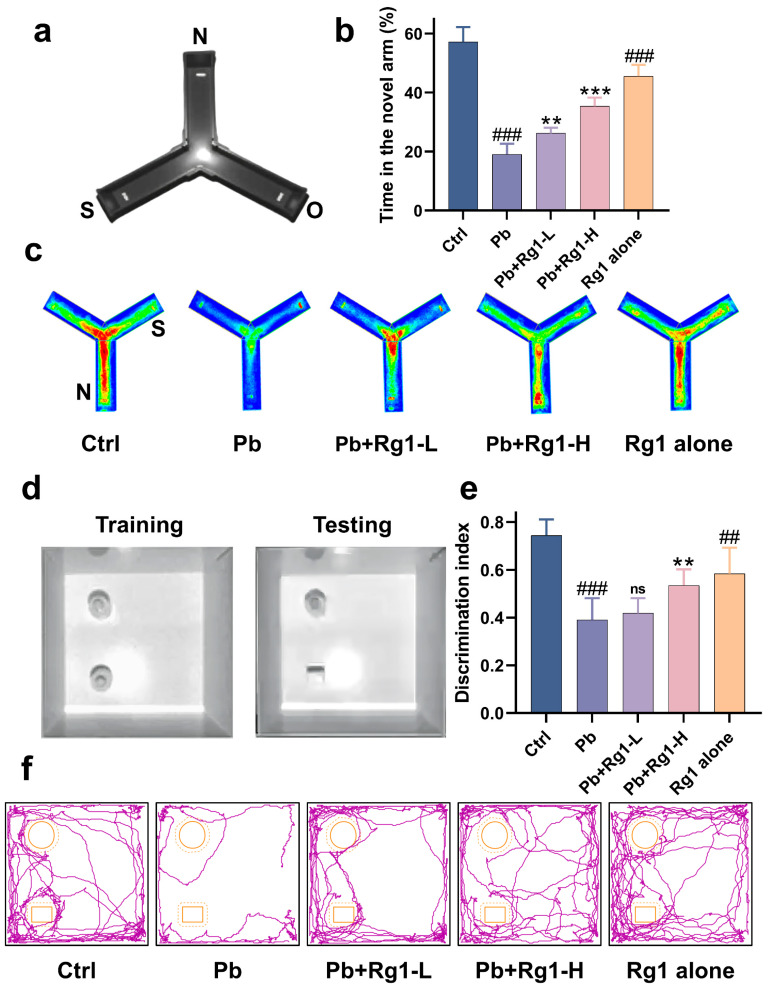
Ginsenoside Rg1 attenuated Pb-induced behavioral deficits in mice. (**a**–**c**) Spatial recognition memory and novel-arm exploration were assessed via the Y-maze test. (**a**) Schematic representation of the Y-maze behavioral apparatus, with S, N, and O denoting the start arm, novel arm, and other arm, respectively. (**b**) Time spent exploring the novel arm, an indicator of spatial recognition memory, with a higher value reflecting a stronger preference for the novel arm. (**c**) Representative Y-maze activity heat maps, in which the color gradient from blue to red represents increasing activity density (**d**–**f**) Novel object recognition (NOR) test. (**d**) Schematic illustration of the training and testing phases. (**e**) Discrimination performance was quantified during the testing phase using the discrimination index, for which higher values denote a greater ability to differentiate the novel object from the familiar one and, consequently, better recognition memory. (**f**) Representative movement trajectories during the test phase of the NOR test. Purple lines indicate the movement trajectories of the mice, while the orange circles and dashed orange squares denote the familiar and novel objects, respectively. Values are expressed as the mean ± SD (*n* = 8 animals in each group). Statistical differences in panels (**b**,**e**) were assessed by one-way ANOVA with Tukey’s post hoc multiple-comparisons test. The Pb and Rg1-alone groups were evaluated relative to the Control group, while the Pb + Rg1 groups were evaluated relative to the Pb group. ## *p* < 0.01 and ### *p* < 0.001 versus the Control group; ** *p* < 0.01 and *** *p* < 0.001 versus the Pb group; ns, not significant.

Following the intervention schedule presented in Figure 1, mice were supplied with drinking water containing 100 ppm lead acetate, while Rg1 was administered by gavage at either a low or high dose. Behavioral performance was subsequently assessed in a fixed sequence, with the Y-maze conducted first, followed by the novel object recognition test and then the Morris water maze, without temporal overlap among the three behavioral paradigms. Rg1 significantly improved Pb-induced deficits in spatial cognition. In the Y-maze task, Pb exposure significantly shortened the duration of novel-arm exploration relative to the Control group (*p* < 0.001; Figure 2a–c). Relative to Pb exposure alone, both Rg1 treatment groups spent more time in the novel arm, and the improvement was more pronounced at the higher dose (*p* < 0.01 for Pb + Rg1-L and *p* < 0.001 for Pb + Rg1-H). During the NOR assessment, Pb exposure markedly reduced the discrimination index relative to the Control group (*p* < 0.001). The Pb-induced decline in the discrimination index was only partially alleviated by Rg1. Specifically, the Pb + Rg1-L group displayed a modest numerical increase that did not reach statistical significance, whereas a significant elevation was detected in the Pb + Rg1-H group versus the Pb group (*p* < 0.01; Figure 2d–f). In contrast, mice in the Rg1-alone group exhibited reduced novel-arm exploration time and a lower discrimination index relative to the Control group (*p* < 0.001 and *p* < 0.01, respectively; Figure 2b,e).

**Figure 3:**
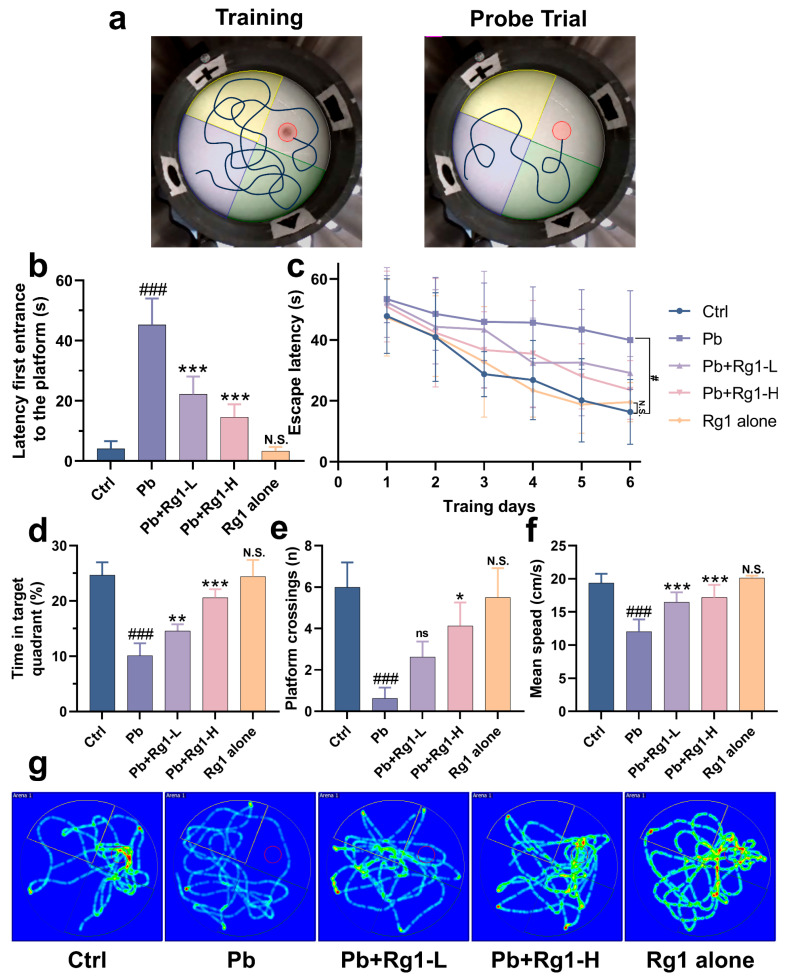
Ginsenoside Rg1 alleviated Pb-evoked deficits in spatial learning and memory in mice. (**a**–**g**) MWM test for evaluating spatial learning and memory. (**a**) Schematic diagram showing the training sessions and probe trial procedure. (**b**) Probe-trial latency to the initial entry into the former platform location. (**c**) Escape latency over the training period. (**d**) Target quadrant residence time during the probe test. (**e**) Probe-trial frequency of crossings over the former platform location. (**f**) Mean swimming speed during the probe trial. (**g**) Representative swimming trajectories during the probe trial. Values are expressed as the mean ± SD (*n* = 8 animals per group). The escape latency in the acquisition phase (**c**) was evaluated using two-way repeated-measures ANOVA with Tukey’s multiple-comparisons test. Statistical analyses for panels (**b**,**d**,**f**) were performed using one-way ANOVA followed by Tukey’s multiple-comparisons test, whereas platform crossing data in panel (**e**) were assessed using the Kruskal–Wallis H test with Dunn’s multiple-comparisons test for post hoc analysis. # *p* < 0.05 and ### *p* < 0.001 versus the Control group; * *p* < 0.05, ** *p* < 0.01, and *** *p* < 0.001 versus the Pb group. Nonsignificant comparisons are denoted according to the corresponding reference group: N.S., not significant versus the Control group; ns, not significant versus the Pb group.

Spatial learning and memory performance was subsequently assessed using the Morris water maze (MWM) test (Figure 3a). Throughout the training phase, all experimental groups showed a gradual reduction in platform-finding latency; however, mice exposed to Pb required more time to locate the concealed platform than control mice (*p* < 0.05), indicating impaired spatial acquisition. Rg1 intervention reduced the prolonged escape latency caused by Pb exposure, with a more evident improvement in the Pb + Rg1-H group (Figure 3c).

During the probe trial of the MWM, Pb-exposed mice showed a prolonged latency to first reach the previous platform site relative to controls (*p* < 0.001; Figure 3b), whereas Rg1 intervention significantly reduced this delay (*p* < 0.001). Impaired spatial memory retention in Pb-exposed mice was further supported by the probe trial results. Relative to Control mice, these animals spent a shorter duration in the target quadrant, made fewer crossings over the previous platform site, and displayed a reduced mean swimming speed (all *p* < 0.001; Figure 3d–f). Relative to the Pb group, target-quadrant residence time was significantly prolonged in the Pb + Rg1-L and Pb + Rg1-H groups (*p* < 0.01 and *p* < 0.001, respectively), while mean swimming speed was significantly elevated in both groups (both *p* < 0.001). Platform crossings also showed a numerical increase in the Pb + Rg1-L group, although this difference was not statistically significant; by contrast, a significant elevation was detected in the Pb + Rg1-H group (*p* < 0.05). Representative swimming trajectories further showed less focused search behavior in Pb-exposed mice, whereas Rg1-treated mice displayed more concentrated search patterns within the target quadrant (Figure 3g). In addition, the Rg1-alone and Control groups performed comparably during both the acquisition phase and the probe trial, with no measured outcome reaching statistical significance (all *p* > 0.05; Figure 3b–f).

### 2.2. Rg1 Attenuated Pb-Induced Hippocampal Neuronal Degeneration and Neuronal Loss

**Figure 4:**
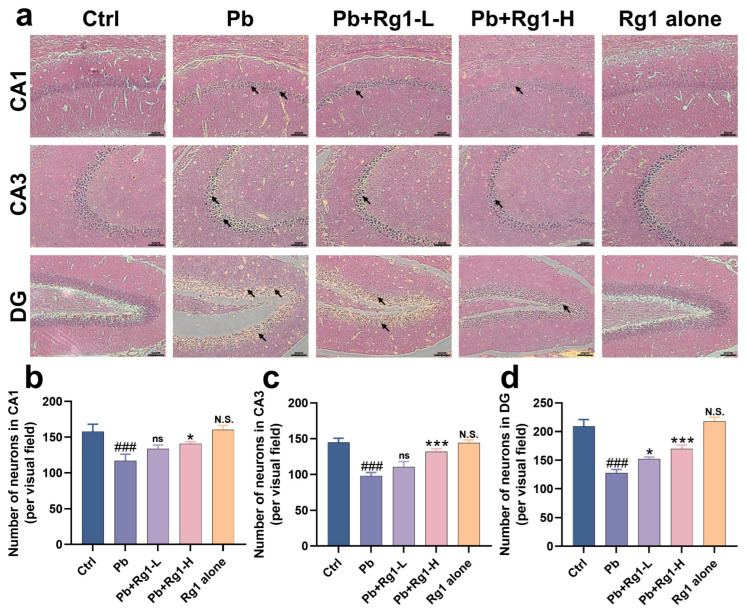
Ginsenoside Rg1 alleviated Pb-evoked hippocampal neuronal degeneration and neuronal depletion in mice. (**a**) Representative H&E-stained sections of the hippocampal CA1, CA3, and dentate gyrus (DG) subfields. Black arrows highlight shrunken neurons characterized by pyknotic nuclei and perineuronal vacuolization. Scale bar: 50 μm. (**b**–**d**) Quantitative analysis of neuronal counts in the CA1, CA3, and DG subfields. Data are presented as the mean ± SD, with *n* = 3 animals per group. Following completion of the behavioral assessments, three of the eight animals in each group were chosen at random before any tissue-based analyses were performed. Selection was conducted independently of all behavioral, biochemical, histological, and molecular findings. Data in (**b**–**d**) were analyzed using one-way ANOVA followed by Tukey’s multiple-comparisons test. ### *p* < 0.001 versus the Control group; * *p* < 0.05 and *** *p* < 0.001 versus the Pb group. Nonsignificant comparisons are denoted according to the corresponding reference group: N.S., not significant versus the Control group; ns, not significant versus the Pb group.

To further determine whether the observed behavioral impairments were accompanied by structural neuronal alterations, H&E staining was conducted to assess hippocampal morphology. Compared with controls, Pb-exposed mice displayed marked pathological abnormalities in the CA1, CA3, and DG subfields, characterized by neuronal atrophy, condensed nuclei, and perineuronal vacuole formation (Figure 4a). Quantitative analysis showed that Pb exposure significantly reduced neuronal counts in the CA1, CA3, and DG subfields compared with the Control group (all *p* < 0.001; Figure 4b–d). Relative to the Pb group, the Pb + Rg1-L group exhibited a numerical elevation in neuronal counts within the CA1 and CA3 regions, although these differences did not achieve statistical significance, whereas a significant increase was detected in the DG region (*p* < 0.05). The Pb + Rg1-H group significantly increased neuronal counts in all three hippocampal subfields, with significance detected in the CA1 region (*p* < 0.05) and more pronounced effects in the CA3 and DG regions (both *p* < 0.001). In addition, no significant differences were detected between the Rg1-alone and Control groups in the CA1, CA3, or DG subfields (all *p* > 0.05), consistent with the preserved hippocampal morphology shown in the representative H&E images (Figure 4a–d).

**Figure 5:**
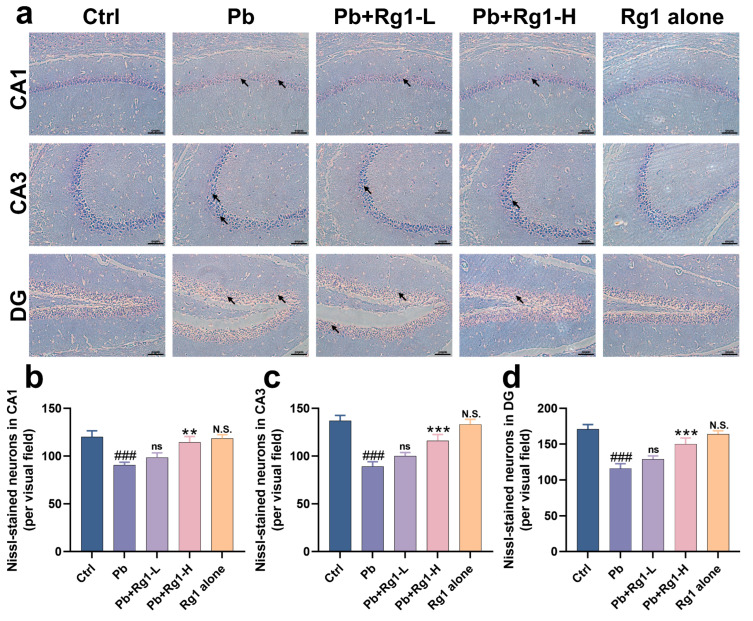
Ginsenoside Rg1 alleviated Pb-evoked reduction of Nissl-positive neurons in the mouse hippocampus. (**a**) Representative Nissl-stained sections of the hippocampal CA1, CA3, and dentate gyrus (DG) subfields. Black arrows indicate degenerative neurons with diminished Nissl bodies and abnormal morphology. (**b**–**d**) Quantitative analysis of Nissl-positive neuronal counts in the CA1, CA3, and DG subfields. Data are presented as the mean ± SD, with *n* = 3 animals per group. Data in (**b**–**d**) were analyzed using one-way ANOVA followed by Tukey’s multiple-comparisons test. ### *p* < 0.001 versus the Control group; ** *p* < 0.01 and *** *p* < 0.001 versus the Pb group. Nonsignificant comparisons are denoted according to the corresponding reference group: N.S., not significant versus the Control group; ns, not significant versus the Pb group.

Nissl staining further supported the neuroprotective action of Rg1 in the hippocampus. Pb exposure markedly decreased the abundance of Nissl-stained neurons and attenuated Nissl staining intensity within the CA1, CA3, and DG subfields (Figure 5a). Quantification demonstrated significantly lower numbers of Nissl-positive neurons in the CA1, CA3, and DG subfields of Pb-exposed mice than in Control mice. (*p* < 0.001; Figure 5b–d). Compared with the Pb group, the Pb + Rg1-L group showed modest numerical increases in Nissl-positive neuronal counts in the CA1, CA3, and DG subfields, although none of the observed changes achieved statistical significance. In contrast, the Pb + Rg1-H group significantly increased Nissl-positive neuronal counts in the CA1 region (*p* < 0.01) and in the CA3 and DG regions (both *p* < 0.001; Figure 5b–d). Likewise, the Rg1-alone group did not differ significantly from the Control group in any of the three hippocampal subfields (all *p* > 0.05), consistent with the representative images showing abundant Nissl-positive neurons, preserved staining intensity, and maintained hippocampal cytoarchitecture in the CA1, CA3, and DG regions (Figure 5a–d). These histological findings further demonstrate that Rg1 not only improved Pb-induced cognitive deficits but also protected hippocampal neurons from Pb-induced damage.

### 2.3. Rg1 Mitigated Pb-Induced Neuroinflammation and Oxidative Stress in Mouse Brain Tissues

**Figure 6:**
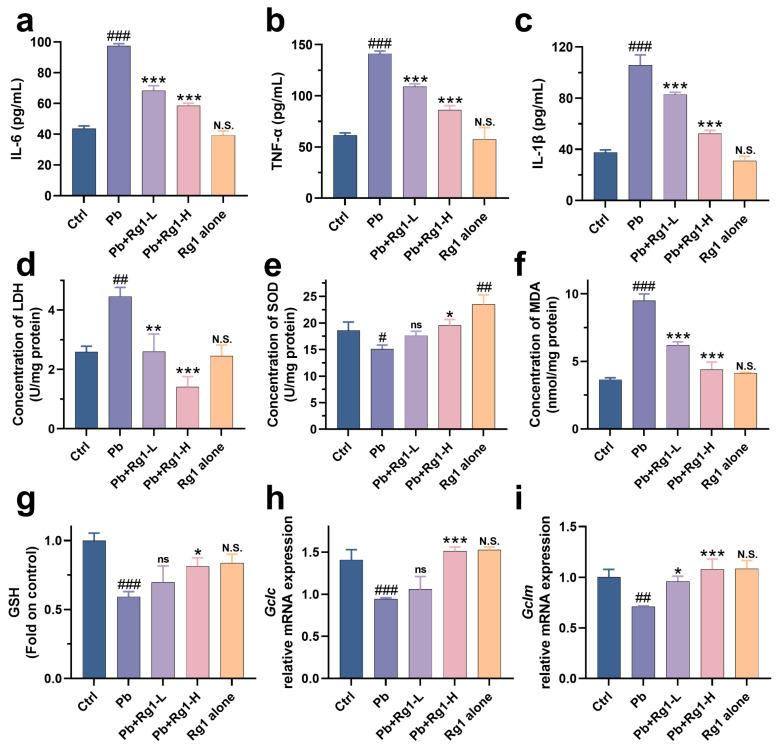
Ginsenoside Rg1 alleviated Pb-evoked neuroinflammatory responses and oxidative injury in mice. (**a**–**c**) Concentrations of pro-inflammatory cytokines in mouse brain tissues: (**a**) interleukin-6 (IL-6), (**b**) tumor necrosis factor-α (TNF-α), and (**c**) interleukin-1β (IL-1β). (**d**–**g**) Biochemical indicators associated with oxidative injury and antioxidant defense in mouse brain tissues: (**d**) lactate dehydrogenase (LDH), (**e**) superoxide dismutase (SOD), (**f**) malondialdehyde (MDA), and (**g**) glutathione (GSH). (**h**,**i**) Relative mRNA expression of *Gclc* and *Gclm*. Data are presented as the mean ± SD, with *n* = 3 animals per group. Data in (**a**–**i**) were analyzed using one-way ANOVA followed by Tukey’s multiple-comparisons test. # *p* < 0.05, ## *p* < 0.01, and ### *p* < 0.001 versus the Control group; * *p* < 0.05, ** *p* < 0.01, and *** *p* < 0.001 versus the Pb group. Nonsignificant comparisons are denoted according to the corresponding reference group: N.S., not significant versus the Control group; ns, not significant versus the Pb group.

To further examine the biochemical changes underlying hippocampal injury, pro-inflammatory cytokines were measured in mouse brain tissues. Pb exposure markedly increased IL-6, TNF-α, and IL-1β levels compared with the Control group (*p* < 0.001) (Figure 6a–c). Rg1 administration significantly lowered the levels of these cytokines, with a more pronounced reduction observed in the high-dose group (*p* < 0.001; Figure 6a–c). In parallel, no significant differences in IL-6, TNF-α, or IL-1β levels were observed between the Rg1-alone and Control groups (all *p* > 0.05; Figure 6a–c).

Oxidative stress-related indicators were then assessed in mouse brain tissues. Pb exposure significantly increased LDH and MDA levels while reducing SOD activity and GSH content relative to the Control group (Figure 6d–g). Compared with the Pb group, the Pb + Rg1 groups significantly reduced LDH and MDA levels, whereas significant increases in SOD activity and GSH content were mainly observed in the Pb + Rg1-H group (Figure 6d–g). At the transcriptional level, *Gclc* expression increased numerically in the Pb + Rg1-L group without reaching statistical significance, while *Gclm* expression was significantly elevated (*p* < 0.05). In contrast, both *Gclc* and *Gclm* expression were significantly increased in the Pb + Rg1-H group relative to the Pb group (both *p* < 0.001; Figure 6h,i). Compared with the Control group, Rg1 alone did not significantly alter LDH, MDA, GSH, *Gclc*, or *Gclm*, but significantly increased SOD activity (*p* < 0.01; Figure 6d–i).

### 2.4. Rg1 Attenuated Pb-Induced Cytotoxic Injury in HT22 Cells In Vitro

**Figure 7:**
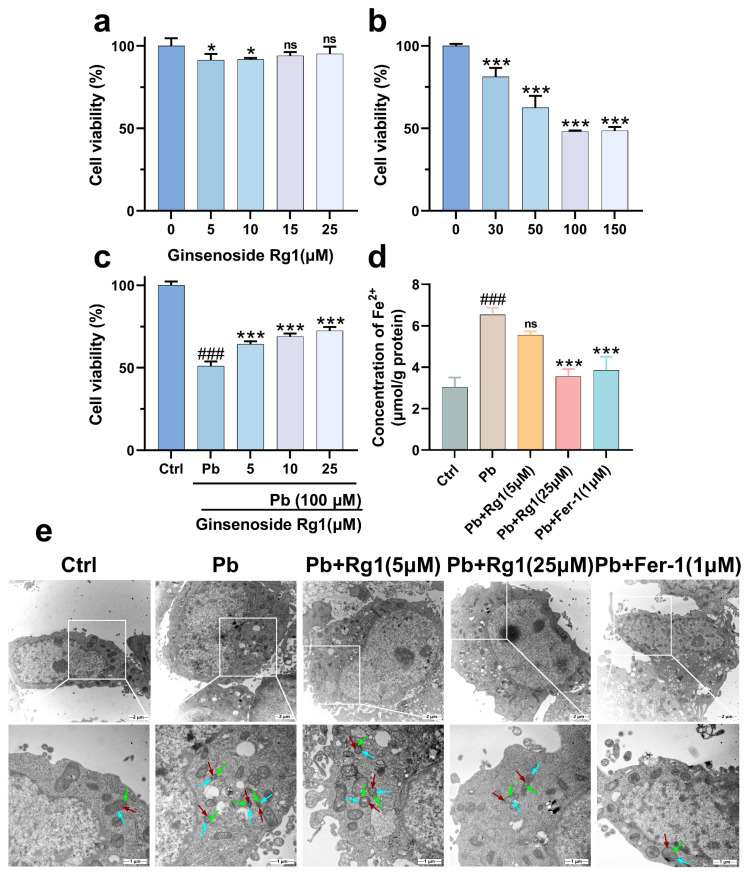
Ginsenoside Rg1 alleviated Pb-evoked cytotoxicity and ferroptosis-associated mitochondrial abnormalities in HT22 cells. (**a**) Effects of increasing concentrations of Rg1 on HT22 cell viability. (**b**) Viability of HT22 cells following exposure to different Pb concentrations. (**c**) Rg1-mediated protection against Pb-evoked cellular injury in HT22 cells. (**d**) Intracellular Fe^2+^ levels in HT22 cells. (**e**) Representative transmission electron microscopy (TEM) images showing ultrastructural mitochondrial alterations in HT22 cells. Enlarged views indicate mitochondrial features marked by colored arrows: red arrows, mitochondrial cristae; green arrows, mitochondrial matrix; and blue arrows, mitochondrial size and membrane density. Scale bars correspond to 2 μm in the low-magnification micrographs and 1 μm in the high-magnification micrographs. Quantitative data are presented as the mean ± SD from three independent biological replicates. Data in (**a**–**d**) were analyzed using one-way ANOVA followed by Tukey’s multiple-comparisons test. In (**a**,**b**), * *p* < 0.05 and *** *p* < 0.001 versus the untreated Control group, respectively; ns in (**a**) indicates no significant difference versus the untreated Control group. In (**c**,**d**), ### *p* < 0.001 versus the Control group and *** *p* < 0.001 versus the Pb group; ns in (**d**) indicates no significant difference versus the Pb group.

To corroborate the in vivo observations, HT22 cells were employed to determine whether Rg1 could directly attenuate Pb-induced cytotoxicity in neuronal cells in vitro. Pb treatment decreased HT22 cell viability in a dose-related manner, with significant decreases detected at 30–100 μM relative to untreated cells (*p* < 0.001) (Figure 7b). Among the concentrations examined, exposure to 100 μM Pb lowered cell viability by nearly 50%. Therefore, this concentration was chosen for subsequent cellular experiments. Rg1 maintained cell viability above 90% at 5–25 μM. Small but significant reductions were observed at 5 and 10 μM (*p* < 0.05), whereas 15 and 25 μM did not differ significantly from untreated cells (Figure 7a). Under 100 μM Pb challenge, Rg1 at 5–25 μM significantly increased cell viability relative to the Pb group (*p* < 0.001; Figure 7c). Accordingly, 100 μM Pb was used to induce cellular injury, whereas 5 and 25 μM Rg1 were designated as the low- and high-concentration interventions for subsequent in vitro experiments.

### 2.5. Rg1 Alleviated Pb-Induced Ferroptosis-Related Alterations in HT22 Cells

Given the prominent oxidative stress observed above, Fe^2+^ accumulation and mitochondrial ultrastructure were further examined to assess ferroptosis-related changes in Pb-exposed HT22 cells. Intracellular Fe^2+^ levels were markedly elevated following Pb exposure relative to the Control group (*p* < 0.001; Figure 7d). The Pb + Rg1 (5 μM) group did not differ significantly from the Pb group, whereas the Pb + Rg1 (25 μM) and Pb + Fer-1 groups showed significantly reduced Fe^2+^ levels (*p* < 0.001; Figure 7d). Fer-1, used at a previously reported concentration [26], also decreased Pb-induced Fe^2+^ accumulation (*p* < 0.001). Consistently, TEM micrographs showed intact mitochondria with clear cristae in Control cells, while Pb-exposed cells displayed shrunken mitochondria, increased membrane density, and disrupted cristae (Figure 7e). These mitochondrial alterations were alleviated after Rg1 treatment, particularly at 25 μM, and a similar morphological improvement was observed in the Fer-1 group. Collectively, these findings suggest that modulation of ferroptosis may contribute to the protective effects of Rg1 against Pb-induced injury in HT22 cells.

### 2.6. Rg1 Restored xCT/GPX4 Expression and Activated Nrf2 Signaling in Mouse Brain Tissues

**Figure 8:**
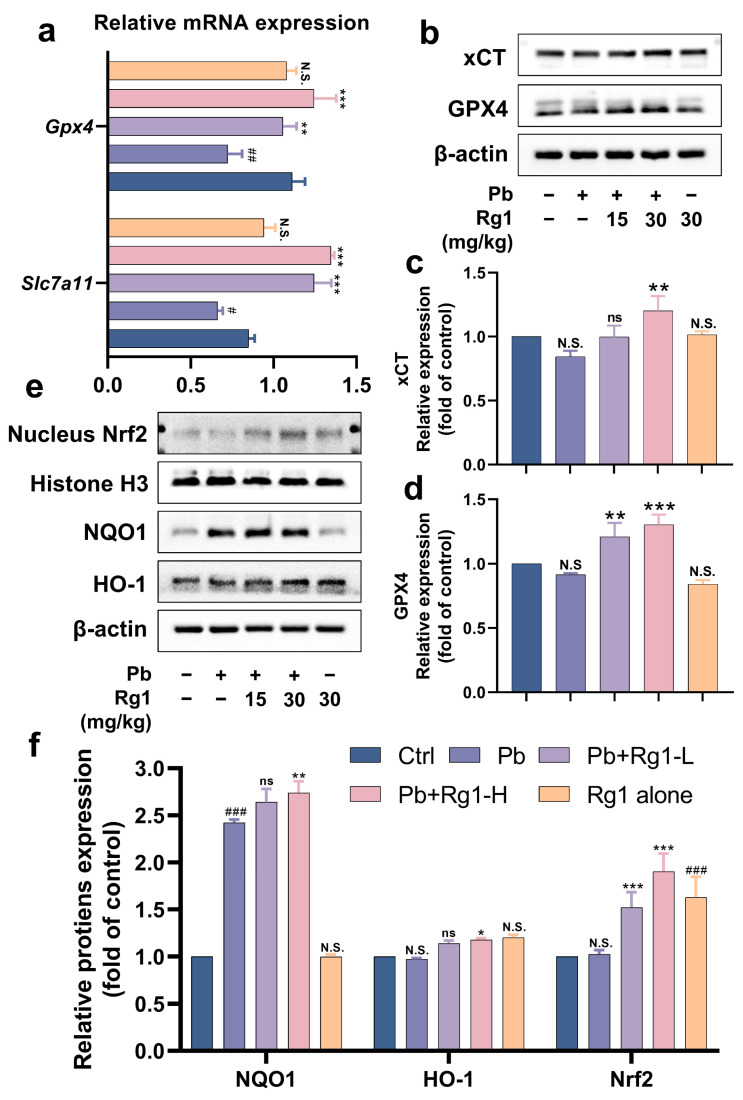
Ginsenoside Rg1 activated the Nrf2 signaling pathway and regulated ferroptosis-related proteins in the mouse brain. (**a**) Relative transcript levels of the ferroptosis-associated genes *Slc7a11* and *Gpx4* in mouse brain tissues. (**b**) Representative Western blot bands of ferroptosis-related proteins in mouse brain tissues, including xCT and GPX4, with β-actin as a loading control. (**c**,**d**) Quantitative analysis of ferroptosis-related protein levels: (**c**) xCT and (**d**) GPX4 expression. (**e**) Representative Western blot bands of Nrf2 signaling pathway-related proteins in mouse brain tissues, including nuclear Nrf2, NQO1, and HO-1. Histone H3 was used as the loading control for nuclear Nrf2, and β-actin was used as the loading control for NQO1 and HO-1. (**f**) Quantification of protein expression levels: NQO1, HO-1, and nuclear Nrf2 expression. Data are presented as the mean ± SD, with *n* = 3 animals per group. Data in (**a**,**d**,**f**) were analyzed using one-way ANOVA followed by Tukey’s multiple-comparisons test, whereas xCT expression in (**c**) was analyzed using the Kruskal–Wallis H test followed by Dunn’s multiple-comparisons test. # *p* < 0.05, ## *p* < 0.01 and ### *p* < 0.001 versus the Control group; * *p* < 0.05, ** *p* < 0.01, and *** *p* < 0.001 versus the Pb group. Nonsignificant comparisons are denoted according to the corresponding reference group: N.S., not significant versus the Control group; ns, not significant versus the Pb group.

Following the ferroptosis-related changes observed in HT22 cells, the xCT/GPX4 axis was further examined in mouse brain tissues. Transcriptional analysis by qRT-PCR indicated that Pb treatment suppressed *Slc7a11* and *Gpx4* mRNA levels, indicating impaired anti-ferroptotic defense (*p* < 0.05 or *p* < 0.001) (Figure 8a). Rg1 treatment markedly restored *Slc7a11* and *Gpx4* mRNA expression, with the Pb+Rg1-H group showing the most pronounced increase (*p* < 0.001). In line with these transcriptional changes, Western blot analysis showed numerical decreases in xCT and GPX4 protein levels following Pb exposure, although neither difference reached statistical significance (Figure 8c,d). Rg1 treatment increased both proteins in a dose-related pattern; however, the increase in xCT expression reached statistical significance only in the Pb + Rg1-H group, whereas GPX4 expression was significantly increased in both Pb + Rg1 groups. Specifically, xCT expression was significantly higher in the Pb + Rg1-H group than in the Pb group (*p* < 0.01; Figure 8c), whereas GPX4 expression was significantly higher in the Pb + Rg1-L and Pb + Rg1-H groups (*p* < 0.01 and *p* < 0.001, respectively; Figure 8d). In addition, neither xCT nor GPX4 expression differed significantly between the Rg1-alone and Control groups (both *p* > 0.05; Figure 8c,d).

The Nrf2 pathway was subsequently assessed to explore the upstream antioxidant response associated with these changes. Pb exposure significantly increased NQO1 expression relative to the Control group (*p* < 0.001; Figure 8f), whereas neither nuclear Nrf2 accumulation nor HO-1 expression differed significantly. Compared with the Pb group, NQO1 expression was significantly elevated in the Pb + Rg1-H group (*p* < 0.01), while no significant change was detected in the Pb + Rg1-L group. Nuclear accumulation of Nrf2 increased significantly in both Pb + Rg1 groups (*p* < 0.001); however, HO-1 expression was significantly increased only following high-dose Rg1 treatment (*p* < 0.05; Figure 8f). Compared with the Control group, Rg1 alone significantly elevated nuclear Nrf2 protein levels (*p* < 0.001), whereas NQO1 and HO-1 expression remained unchanged. These findings indicate that Rg1 strengthened xCT/GPX4-mediated anti-ferroptotic defense in Pb-exposed mouse brain tissues. These changes were accompanied by activation of Nrf2 signaling, supporting the possible involvement of this pathway.

### 2.7. Nrf2 Inhibition Partially Attenuated the Anti-Ferroptotic Effect of Rg1 in Pb-Exposed HT22 Cells

**Figure 9:**
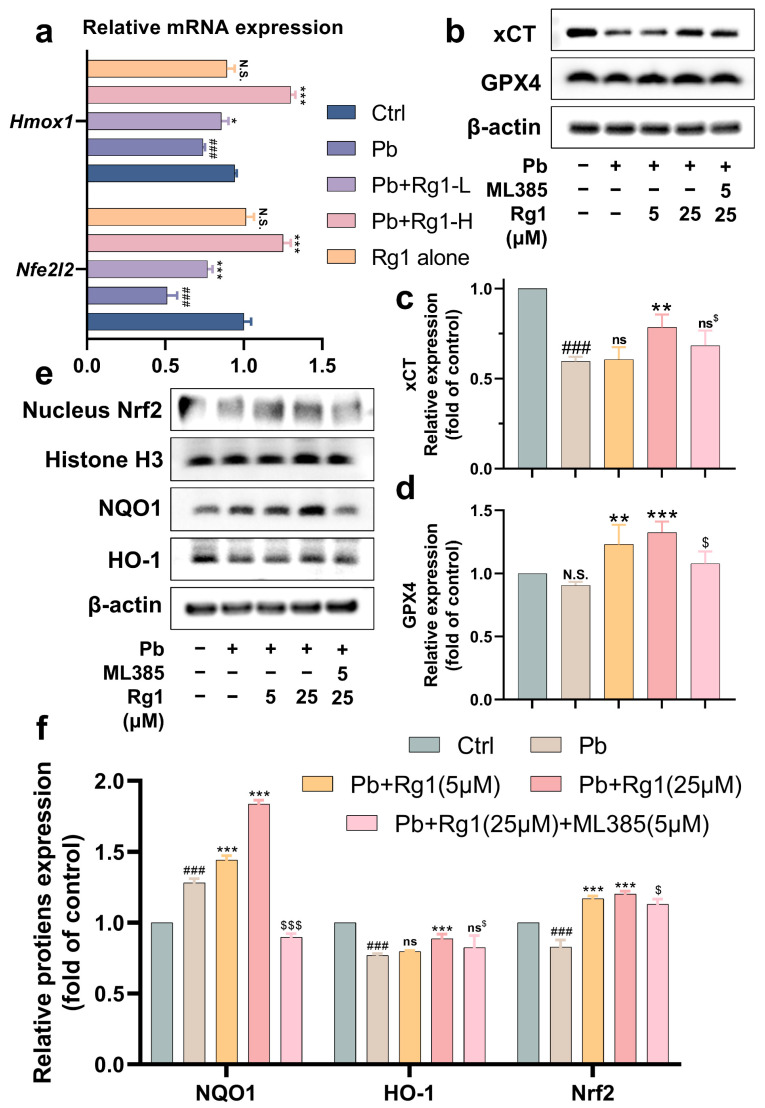
Nrf2 inhibition partially attenuated the anti-ferroptotic effects of ginsenoside Rg1 in Pb-exposed HT22 cells. (**a**) Relative mRNA expression levels of Nrf2 pathway-related genes in mouse brain tissues, including *Hmox1* and *Nfe2l2*. (**b**) Representative Western blot bands of ferroptosis-related proteins in HT22 cells, including xCT and GPX4, with β-actin as a loading control. (**c**,**d**) Quantitative analysis of protein levels: (**c**) xCT and (**d**) GPX4 expression. (**e**) Representative Western blot bands of Nrf2 pathway-associated proteins in HT22 cells, including nuclear Nrf2, NQO1, and HO-1. Histone H3 served as the loading control for nuclear Nrf2, whereas β-actin was used as the internal control for NQO1 and HO-1. (**f**) Quantification of NQO1, HO-1, and nuclear Nrf2 protein expression. Data are presented as the mean ± SD. In (**a**), *n* = 3 animals per group; in (**c**,**d**,**f**), *n* = 3 independent biological replicates. Data were analyzed using one-way ANOVA followed by Tukey’s multiple-comparisons test. ### *p* < 0.001 versus the Control group; * *p* < 0.05, ** *p* < 0.01, and *** *p* < 0.001 versus the Pb group; $ *p* < 0.05 and $$$ *p* < 0.001 versus the Pb + Rg1 (25 μM) group. Nonsignificant comparisons are denoted according to the corresponding reference group: N.S., versus the Control group; ns, versus the Pb group; and ns^$^, versus the Pb + Rg1 (25 μM) group.

To clarify whether Nrf2 signaling contributes to the anti-ferroptotic action of Rg1, Nrf2-associated genes were initially assessed in mouse brain tissues, and mechanistic validation was subsequently performed in HT22 cells using the Nrf2 inhibitor ML385. At the transcriptional level, Pb exposure downregulated *Nfe2l2* and *Hmox1* expression relative to the Control group (*p* < 0.001) (Figure 9a). Rg1 treatment increased both *Nfe2l2* and *Hmox1* expression, with the Pb+Rg1-H group showing the strongest response (*p* < 0.001). In contrast, neither transcript differed significantly between the Rg1-alone and Control groups (both *p* > 0.05; Figure 9a).

We next investigated whether Nrf2 signaling contributed to the protective effects of Rg1, with ML385 introduced into Pb-exposed HT22 cells at a concentration selected from previous studies [27]. Pb exposure significantly decreased xCT expression relative to the Control group (*p* < 0.001) (Figure 9c), while 25 μM Rg1 increased xCT expression relative to the Pb group (*p* < 0.01). However, ML385 did not significantly reduce xCT expression compared with the Pb + Rg1 (25 μM) group. For GPX4, both 5 and 25 μM Rg1 significantly increased its expression relative to the Pb group (*p* < 0.01 and *p* < 0.001, respectively; Figure 9d), with a larger numerical increase observed at 25 μM. Notably, compared with the Pb + Rg1 (25 μM) group, co-treatment with ML385 led to a partial decline in GPX4 protein levels, suggesting that Nrf2 inhibition weakened the Rg1-mediated upregulation of GPX4 (*p* < 0.05). Consistently, 25 μM Rg1 markedly increased NQO1, HO-1, and nuclear Nrf2 expression in Pb-exposed HT22 cells (*p* < 0.001) (Figure 9e,f). ML385 significantly decreased NQO1 and nuclear Nrf2 expression compared with the Pb + Rg1 (25 μM) group (*p* < 0.001 and *p* < 0.05) (Figure 9f), whereas HO-1 expression did not differ significantly between the Pb + Rg1 (25 μM) + ML385 and Pb + Rg1 (25 μM) groups. These results indicate that ML385 partially weakened the effects of Rg1 on nuclear Nrf2, NQO1, and GPX4 expression in Pb-exposed HT22 cells. In contrast, xCT and HO-1 expression remained unchanged. These findings support the involvement of Nrf2 signaling but also suggest that additional pathways may contribute to the protective effect of Rg1.

## 3. Discussion

The present study provides systematic evidence that ginsenoside Rg1 alleviates Pb-induced cognitive impairment and neuronal damage, potentially through the attenuation of inflammatory responses, oxidative stress, and ferroptosis-associated injury. The main findings are as follows: (1) Rg1 improved hippocampus-dependent cognitive function; (2) attenuated hippocampal neuronal injury and inflammatory and oxidative responses; (3) reduced Pb-induced neuronal ferroptosis; and (4) provided pharmacological evidence supporting the involvement of Nrf2 signaling in the anti-ferroptotic effect of Rg1. Together, these findings provide experimental evidence that Rg1 attenuates Pb-induced neurotoxicity and support further preclinical investigation of its underlying neuroprotective mechanisms.

Pb-induced cognitive dysfunction is commonly accompanied by hippocampal structural damage, consistent with the central role of the hippocampus in learning and memory [9]. Thus, preservation of hippocampal neuronal integrity represents a critical aspect of protection against Pb-related neurotoxicity. Although Rg1 has been reported to exert neuroprotective activity [23], its efficacy in Pb-induced cognitive and hippocampal injury remains unclear. In this study, Rg1 improved hippocampus-dependent cognitive performance in multiple behavioral tests, including the Y-maze, NOR, and MWM. These effects were supported by H&E and Nissl staining, which showed preserved neuronal morphology and reduced neuronal loss in the CA1, CA3, and DG regions. Interestingly, under physiological conditions, Rg1 administration alone reduced novel-arm exploration and the NOR discrimination index, whereas no significant changes were detected in MWM performance or hippocampal neuronal counts. This dissociation may reflect a task-specific influence on novelty-directed exploration or recognition-related behavior rather than generalized learning and memory impairment or overt hippocampal injury. Nevertheless, potential effects of Rg1 on exploratory motivation or novelty preference cannot be excluded. Importantly, Rg1 improved the corresponding behavioral deficits and preserved hippocampal neuronal integrity under Pb exposure. Collectively, these findings suggest that Rg1 attenuates Pb-induced hippocampus-dependent cognitive impairment and neuronal loss. Although male mice were selected to maintain a relatively homogeneous experimental cohort and reduce variability associated with sex-specific physiological factors, previous studies have reported sex-dependent differences in the behavioral and neurobiological effects of Pb exposure [28,29]. Therefore, whether Rg1 provides comparable neuroprotection in female mice remains to be determined. Nevertheless, the observed improvements in behavioral performance and histological outcomes led us to further explore the mechanisms responsible for the protective action of Rg1.

Oxidative stress and neuroinflammation are important pathological features of Pb-induced neurotoxicity [30,31]. In the present study, Pb exposure increased IL-6, TNF-α, IL-1β, LDH, and MDA levels, while reducing SOD activity, GSH content, and *Gclc*/*Gclm* expression. Rg1 markedly reduced inflammatory cytokine release and oxidative injury markers, while restoring antioxidant defenses and GSH-related gene expression. These effects may help re-establish redox balance and limit inflammatory damage under Pb exposure. Considering that reduced GSH availability and enhanced lipid peroxidation are central biochemical changes that increase cellular susceptibility to ferroptosis [32,33], ferroptosis-related injury was therefore considered a downstream consequence of Pb-induced redox imbalance.

Ferroptosis represents an iron-dependent form of regulated cell death, primarily driven by excessive lipid peroxidation and impairment of cellular antioxidant defense mechanisms [34]. This process is highly relevant to neuronal injury because neural cells are vulnerable to iron dyshomeostasis and oxidative lipid damage. In the context of Pb neurotoxicity, previous studies have reported that Pb exposure disrupts iron homeostasis and weakens ferroptosis-related defense in neural cells [35,36]. Consistent with this evidence, Pb exposure increased Fe^2+^ accumulation in HT22 cells and induced mitochondrial shrinkage, increased membrane density, and cristae disruption. Rg1 alleviated these mitochondrial alterations, especially at the high concentration, and Fer-1 produced a similar protective pattern. At the molecular level, Pb impaired ferroptosis-related defense, as shown by reduced *Slc7a11*/*Gpx4* expression and decreased xCT/GPX4 protein levels in mouse brain tissues. Rg1 restored these changes, with a more evident effect on GPX4 expression. These results suggest that Rg1 limits Pb-induced neuronal ferroptosis, possibly by preserving GPX4-centered anti-ferroptotic defense.

The restoration of GPX4-centered anti-ferroptotic defense further pointed to the involvement of Nrf2 signaling. Nrf2 is a key regulator of redox homeostasis, inflammatory responses, and ferroptosis susceptibility [37]. Upon activation, Nrf2 accumulates in the nucleus and promotes cytoprotective responses through downstream proteins such as HO-1 and NQO1, which are closely associated with antioxidant defense and cellular stress resistance [38]. In the present study, Rg1 promoted nuclear Nrf2 accumulation and increased NQO1 and HO-1 expression in mouse brain tissues. Rg1 also upregulated *Nfe2l2* and *Hmox1* mRNA levels, indicating activation of Nrf2-associated antioxidant signaling. Under physiological conditions, Rg1 alone increased SOD activity and nuclear Nrf2 accumulation without significantly altering the other inflammatory, oxidative stress-related, ferroptosis-related, or Nrf2-associated endpoints. This selective response may reflect modulation of basal antioxidant defense rather than a broad disturbance of redox or ferroptosis-related homeostasis. To further assess the role of this pathway, ML385 was applied in Pb-exposed HT22 cells. ML385 weakened the effects of Rg1 on nuclear Nrf2 and NQO1 expression and partially reduced GPX4 expression. In contrast, xCT and HO-1 were not significantly affected. These findings support a contributory role for Nrf2 signaling in Rg1-mediated anti-ferroptotic protection, particularly in relation to GPX4-associated defense. However, the incomplete inhibitory effect of ML385 and the unchanged xCT and HO-1 levels indicate that Nrf2 is unlikely to be the sole mediator. Previous studies have identified additional pathways underlying the neuroprotective effects of Rg1. Huang et al. reported that Rg1 enhanced Akt and ERK1/2 phosphorylation in Aβ25–35-exposed hippocampal neurons, whereas inhibition of either pathway attenuated its effects on neurite outgrowth and neuronal survival [39]. Wang et al. further showed that Rg1 improved mitochondrial function and cognitive deficits in APP/PS1 mice and Aβ1–42-treated HT22 cells through miR-9-5p/SIRT1 signaling, with these effects weakened by SIRT1 inhibition [40]. These findings suggest that Akt/ERK1/2 and SIRT1 signaling may act alongside Nrf2 and partly account for the residual protective effects observed after ML385 treatment. Their roles in Pb-induced neurotoxicity require further investigation.

Several limitations should be acknowledged. First, Pb concentrations in blood and brain tissues were not measured, which limits the assessment of whether Rg1 also affects Pb bioavailability or tissue distribution. Second, Nrf2 involvement was assessed only using the pharmacological inhibitor ML385. Genetic loss-of-function approaches are required to determine whether Nrf2 is necessary for Rg1-mediated protection and to establish pathway causality. Third, ferroptosis was mainly assessed by Fe^2+^ accumulation, mitochondrial morphology, and xCT/GPX4 expression. Further analysis of iron metabolism and lipid peroxidation-related markers is needed. Fourth, the cellular specificity of Rg1-mediated protection across different hippocampal cell populations remains to be clarified. These issues require further investigation.

## 4. Methods and Materials

### 4.1. Reagents

Ginsenoside Rg1 (purity ≥ 98%) and lead acetate [Pb(Ac)_2_, purity > 98%] were purchased from Chengdu Must Bio-Technology Co., Ltd. (Chengdu, China). ML385 (>99%), a selective Nrf2 inhibitor, was obtained from Yuanye Bio-Technology (Shanghai, China), whereas ferrostatin-1 (Fer-1) was supplied by Aladdin (Shanghai, China). The CCK-8 kit and ferrous ion assay kit were obtained from Solarbio (Beijing, China). Commercial assay kits for malondialdehyde (MDA), total glutathione/oxidized glutathione (T-GSH/GSSG), total superoxide dismutase (T-SOD), and lactate dehydrogenase (LDH) were supplied by Nanjing Jiancheng Bioengineering Institute (Nanjing, China). All other chemicals and reagents used in this study were of analytical grade unless otherwise indicated.

### 4.2. Animals

Three-week-old male C57BL/6J mice weighing 10–12 g were supplied by Liaoning Changsheng Biotechnology Co., Ltd. (Benxi, China) [SCXK (Liao) 2020-0001]. All experimental procedures involving animals were reviewed and approved by the Animal Ethics Committee of Jilin University (Approval No. YNPZSY2024066).

After 1 week of acclimatization, the mice were randomly assigned to five experimental groups, with eight animals per group: (1) normal control group; (2) Pb-exposed model group; (3) Pb + Rg1 group receiving 15 mg/kg Rg1; (4) Pb + Rg1 group receiving 30 mg/kg Rg1; and (5) Rg1 alone group receiving 30 mg/kg Rg1. Mice in groups 2–4 were exposed to Pb via drinking water containing 100 ppm Pb(Ac)_2_ for 3 months to establish a Pb-induced neurotoxicity model, as previously described. In addition, mice in groups 3–5 were administered Rg1 at 15 or 30 mg/kg once daily by oral gavage, with both doses selected based on previous in vivo studies [41].

Predefined humane endpoint criteria were applied throughout the animal experiment. Mice were considered to have reached the endpoint if they showed a body weight reduction exceeding 20% of the baseline value, severe behavioral disturbances, or apparent distress symptoms, including dyspnea and unresponsiveness. After the behavioral tests were completed, the animals were euthanized by cervical dislocation. Brain samples were immediately collected, after which the bilateral hippocampi were carefully separated, rapidly frozen in liquid nitrogen, and stored at −80 °C for subsequent analyses.

### 4.3. Behavioral Assessments

Behavioral assessments were conducted sequentially after the 12-week intervention, with no temporal overlap among the individual paradigms. The Y-maze test was performed first, followed by the NOR test after a 24 h interval. The MWM test was initiated 24 h after completion of the NOR test and continued for seven consecutive days. The specific procedures and experimental phases of each behavioral test are described below.

#### 4.3.1. Y-Maze Assay

Spatial memory was evaluated using a Y-maze apparatus measuring 30 × 7 × 15 cm. In the training session, one arm of the maze was blocked, and each mouse was allowed to freely explore the other two arms for 5 min. After a 2 h interval, the previously closed novel arm was opened, and the mice were placed back into the maze to explore all three arms for another 5 min. Smart 3.0 software was used to track and quantify the exploration duration and travel distance in the novel arm.

#### 4.3.2. Novel Object Recognition (NOR) Assay

The NOR assay was conducted in a square open-field apparatus measuring 40 × 40 × 30 cm. Before the formal experiment, mice were allowed to adapt to the empty arena for 15 min. During the training session, two identical objects were positioned in the arena, and each mouse was allowed to explore freely for 5 min. Following a 24 h interval, one familiar object was substituted with a novel object before the mouse was reintroduced into the arena for testing. The exploration time for each object was recorded. The apparatus and objects were cleaned with 75% ethanol after each trial to minimize olfactory interference. Behavioral data were analyzed using Smart 3.0 software. The discrimination index was calculated as $T_{N}/(T_{N}+T_{F})$, where $T_{N}$ and $T_{F}$ represent the time spent exploring the novel and familiar objects during the testing session, respectively. A higher discrimination index indicates a stronger preference for the novel object and better recognition memory.

#### 4.3.3. Morris Water Maze (MWM) Assay

Spatial learning and memory were assessed using the MWM assay in a circular pool measuring 1.5 m in diameter. A platform was submerged beneath the water surface in the third quadrant. During the 6-day acquisition phase, mice were trained to locate the hidden platform, with a maximum trial duration of 60 s. If a mouse failed to locate the platform within 60 s, the escape latency was recorded as 60 s, and the mouse was gently guided to the platform and allowed to remain there for 30 s. On the seventh day, the platform was removed for the probe test. Escape latency, platform-crossing frequency, and time spent in the target quadrant were recorded for analysis. Mean swimming speed was also recorded to assess locomotor performance.

### 4.4. HE and Nissl Staining

Brain samples were immersed in 10% formalin for 24 h and then subjected to routine paraffin embedding. Sections of 4 μm thickness were prepared from paraffin blocks, followed by deparaffinization and rehydration prior to staining. For hematoxylin and eosin (H&E) staining, tissue sections were incubated with hematoxylin for 5 min and counterstained with eosin for 2 min before mounting. For Nissl staining, sections were stained with 0.5% toluidine blue at 37 °C for 30 min and subsequently mounted. Histopathological changes and neuronal integrity in the hippocampal CA1, CA3, and DG subfields were observed and photographed using an optical microscope (Olympus NV, Aartselaar, Belgium).

### 4.5. Enzyme-Linked Immunosorbent Assay

Brain tissues were homogenized in ice-cold PBS, and the concentrations of TNF-α, IL-6, and IL-1β in the resulting tissue homogenates were determined using the corresponding commercial ELISA kits according to the manufacturers’ instructions. Cytokine concentrations were expressed as pg/mL. Briefly, 50 μL of each standard solution or serum sample diluted at a 1:1 ratio was added to 96-well plates pre-coated with specific monoclonal antibodies. After incubation with horseradish peroxidase (HRP)-labeled detection antibodies, color development was performed using TMB substrate and then stopped. Absorbance was recorded at 450 nm with an automated microplate reader (Tecan Spark, Grödig, Austria). Cytokine concentrations were calculated from the corresponding standard curves.

### 4.6. Biochemical Analysis

Hippocampal samples were homogenized in ice-cold PBS, and the concentrations of lactate dehydrogenase (LDH), malondialdehyde (MDA), total glutathione/oxidized glutathione (T-GSH/GSSG), and total superoxide dismutase (T-SOD) were measured by the corresponding commercial assay kits.

### 4.7. Cell Culture and Cell Viability Assay

HT22 cells were cultured as previously described [42]. For the CCK-8 assay, cells were seeded into 96-well plates at a density of 4000 cells per well and allowed to adhere overnight. Cell viability was assessed after treatment with different concentrations of Rg1, Pb(Ac)_2_, or their combination. Based on the CCK-8 dose–response results, 100 μM Pb(Ac)_2_ was selected to induce cellular injury, while 5 and 25 μM Rg1 were selected as the low and high concentrations, respectively, for subsequent experiments. The cells were then assigned to the following groups: Control, Pb(Ac)_2_, Pb(Ac)_2_ + Rg1 (5 μM), Pb(Ac)_2_ + Rg1 (25 μM), Pb(Ac)_2_ + Rg1 (25 μM) + ML385 (5 μM), and Pb(Ac)_2_ + Fer-1 (1 μM). After the designated treatments, CCK-8 solution was added to each well, followed by incubation for 2 h. Absorbance was measured at 450 nm.

### 4.8. Ferrous Ion (Fe^2+^) Content Assay

Intracellular Fe^2+^ concentrations were quantified using a ferrous ion assay kit (Beijing Solarbio Science & Technology Co., Ltd., Beijing, China) following the manufacturer’s instructions. Briefly, treated HT22 cells were harvested, disrupted by sonication on ice, and centrifuged at 10,000× *g* for 10 min at 4 °C. The obtained supernatants were collected for Fe^2+^ determination. Absorbance was recorded at 593 nm with a microplate reader, and Fe^2+^ concentrations were calculated according to the corresponding standard curve.

### 4.9. Transmission Electron Microscopy (TEM)

Following the indicated treatments, HT22 cells were harvested by centrifugation and immersed in electron microscopy fixative. For ultrastructural analysis, the samples were processed through sequential post-fixation, dehydration, embedding, sectioning, and contrast staining procedures. Briefly, fixed cell samples were treated with 2% osmium tetroxide, followed by dehydration in a graded ethanol series and embedding in Epon 812 resin. Ultrathin sections were prepared using an ultramicrotome (Leica Microsystems GmbH, Wetzlar, Germany) and stained with uranyl acetate and lead citrate before observation using a transmission electron microscope (Hitachi High-Tech Corporation, Tokyo, Japan). Mitochondrial morphology was assessed from TEM images.

### 4.10. Western Blotting

For Western blotting, total proteins were extracted from hippocampal samples and HT22 cells using RIPA lysis buffer containing appropriate protease inhibitors. Protein concentrations were then measured with a BCA protein assay kit. Equal amounts of protein were loaded onto 10% SDS-PAGE gels for electrophoretic separation and subsequently transferred onto PVDF membranes. The membranes were blocked with a rapid blocking reagent at room temperature for 1 h, followed by overnight incubation at 4 °C with the primary antibodies listed in Table S1. After washing with TBST, HRP-labeled secondary antibodies were applied for further incubation. The resulting immunoreactive bands were visualized using a gel imaging system (Syntyos, Cambridge, MA, USA), and band densities were analyzed with ImageJ software 1.54p.

### 4.11. Quantitative Real-Time Polymerase Chain Reaction (qRT-PCR)

Total RNA was isolated from mouse brain tissues using the TransZol Up Kit (TransGen Biotech, Beijing, China) and subsequently quantified. According to the manufacturer’s protocol, complementary DNA (cDNA) was synthesized from the extracted RNA using Hieff III 1st Strand cDNA Synthesis SuperMix. Quantitative PCR amplification was carried out on a Bio-Rad CFX96 Touch Deep Well Real-Time PCR Detection System (Bio-Rad, Hercules, CA, USA) using Hieff qPCR SYBR Green Master Mix. The primer sequences applied for RT-qPCR are listed in Table S2.

### 4.12. Statistical Analysis

Results are expressed as the mean ± SD. The Shapiro–Wilk test was used to evaluate data normality, whereas variance homogeneity was examined using the Brown–Forsythe test. Datasets meeting both assumptions were subjected to one-way analysis of variance, with Tukey’s multiple-comparisons test applied for post hoc comparisons. Owing to the relatively small sample sizes and the non-normal distribution of certain datasets, variables that failed the normality test were evaluated using the Kruskal–Wallis H test followed by Dunn’s multiple-comparisons test. Escape latency recorded across the six-day Morris water maze acquisition phase was assessed by two-way repeated-measures analysis of variance, with treatment group and training day included as factors, followed by Tukey’s multiple-comparisons test. All statistical procedures were conducted using GraphPad Prism 10.1.2, and differences were considered statistically significant at *p* < 0.05.

## 5. Conclusions

In summary, this study demonstrates that Rg1 alleviates Pb-induced neurotoxicity by attenuating oxidative stress and ferroptosis-related injury. Evidence from pharmacological inhibition additionally indicates that Nrf2 signaling contributes to these protective effects. Given the preclinical nature of the present study, these findings support further mechanistic and in vivo investigation of Rg1 in Pb-induced neuronal injury. Future studies should define the precise molecular mechanisms of Rg1 action, particularly the contribution of Nrf2-related anti-ferroptotic defense, validate pathway causality using genetic approaches, and confirm the reproducibility of these findings in both sexes and additional Pb-exposure models.

## Data Availability

Data will be made available on request.

## Supplementary Materials

The following supporting information can be downloaded at https://www.mdpi.com/article/10.3390/molecules31152559/s1, Table S1: Primary antibodies used for Western blotting; Table S2: Quantitative Real-time PCR primer sequence list.
