# Supplementary material for: Ginsenoside Rg1 Alleviates Lead-Induced Neurotoxicity Through Nrf2-Associated Modulation of Oxidative Stress and Ferroptosis
Source: Molecules. 2026 Jul 23;31(15):2559. doi: 10.3390/molecules31152559 (PMC13468199; doi:10.3390/molecules31152559)
Supplement: Supplementary file 1 [file molecules-31-02559-s001.zip › molecules-4400337-supplementary.pdf]

Supplementary Material

Table S1. Primary antibodies used for Western blotting.

Primary antibody	Manufacturer	Molecular weight (kDa)	Dilution
xCT	Nature Biosciences, Hangzhou, China	55	1:2000
GPX4	Nature Biosciences, Hangzhou, China	17	1:2000
HO-1	Wanleibio, Shenyang, China	32	1:2000
NQO1	Wanleibio, Shenyang, China	30	1:1000
β-Actin	Proteintech, Wuhan, China	42	1:10000
Nrf2	Nature Biosciences, Hangzhou, China	110	1:2000
Histone H3	Nature Biosciences, Hangzhou, China	15	1:1000

Table S2. Quantitative Real-time PCR primer sequence list.

Gene	Sequences	
Slc7a11	Forward	5'- AGGGCATACTCCAGAACACG -3'
	Reverse	5'- GGACCAAAGACCTCCAGAATG -3'
Gpx4	Forward	5'- GATGGAGCCCATTCCTGAACC -3'
	Reverse	5'- CCCTGTACTTATCCAGGCAGA -3'
Gclc	Forward	5'- CTACCACGCAGTCAAGGACC -3'
	Reverse	5'- CCTCCATTCAGTAACAACTGGAC -3'
Gclm	Forward	5'- CGCCTCCGATTGAAGATGGA -3'
	Reverse	5'- ATTGGGTTTTACCTGTGCCC -3'
Nfe2l2	Forward	5'- CCTCACCTCTGCTGCAAGTA -3'
	Reverse	5'- TCAAATCCATGTCCTGCTGGG -3'
Hmox1	Forward	5'- CAGAGCCGTCTCGAGCATAG -3'
	Reverse	5'- AAATCCTGGGGCATGCTGTC -3'
